# Preoperative systemic inflammatory indices as risk factors for hemorrhage after supratentorial brain tumor resection in patients requiring prolonged neurosurgical intensive care: development and temporal validation of an early postoperative risk stratification model

**DOI:** 10.3389/fonc.2026.1795177

**Published:** 2026-07-20

**Authors:** Biwu Wu, Lichao Wei, Haoyue Yuan, Yu Guo, Jin Hu, Gang Wu

**Affiliations:** 1 Department of Neurosurgical Intensive Care Unit, Huashan Hospital, Fudan University, Shanghai, China; 2 Department of Neurosurgery, Huashan Hospital, Fudan University, Shanghai, China; 3 National Center for Neurological Disorders, Shanghai, China; 4 Shanghai Key Laboratory of Brain Function Restoration and Neural Regeneration, Shanghai, China; 5 Neurosurgical Institute of Fudan University, Shanghai, China; 6 Shanghai Clinical Medical Center of Neurosurgery, Shanghai, China

**Keywords:** neurosurgical intensive care, NLR, PLR, postoperative hemorrhage, risk stratification, SIRI, supratentorial brain tumor, systemic inflammation

## Abstract

**Background:**

Postoperative hemorrhage (POH) is a serious complication after supratentorial brain tumor resection. In patients requiring prolonged neurosurgical intensive care unit (NICU) management, effective risk stratification for POH is clinically important but remains limited. Systemic inflammation, quantified by the neutrophil-to-lymphocyte ratio (NLR), platelet-to-lymphocyte ratio (PLR), and systemic inflammation response index (SIRI), has been implicated in hemorrhagic risk. This study aimed to evaluate the associations of NLR, PLR, and SIRI with POH and to develop an integrated early postoperative risk stratification model.

**Methods:**

In this retrospective cohort study, patients with > 48 hours of NICU stay after elective supratentorial tumor resection were temporally divided into development and validation cohorts. Preoperative NLR, PLR, and SIRI were derived from routine blood tests and assessed for associations with POH. Independent risk factors were identified using multivariable logistic regression. The incremental value of inflammatory markers beyond a clinical model was evaluated. Model performance was assessed using receiver operating characteristic analysis, calibration, and decision curve analysis.

**Results:**

The study included 587 patients (438 in development, 149 in validation). POH occurred in 32.9% (144/438) and 32.2% (48/149), respectively. In multivariable analysis, SIRI (adjusted odds ratio [aOR] 1.56, 95% CI 1.18–2.06) and PLR (aOR 1.49 per 100-unit increase, 95% CI 1.05–2.11), together with basal ganglia location, tumor diameter, and postoperative systolic blood pressure (SBP), were independent risk factors for POH; NLR was not. SIRI showed the highest AUC (0.686) compared with NLR (0.673) and PLR (0.669). Adding SIRI to the clinical baseline model provided significant incremental value (IDI 0.043, *P* < 0.001; event NRI 0.208, *P* = 0.011). The final early postoperative model combining SIRI, PLR, and clinical variables achieved an AUC of 0.761 (95% CI 0.716–0.807) in development and 0.731 (95% CI 0.645–0.818) in temporal validation.

**Conclusions:**

Preoperative SIRI and PLR are independent risk factors for POH in patients requiring prolonged NICU stay after supratentorial tumor resection. An integrated early postoperative model combining SIRI and PLR may facilitate risk stratification.

## 1 Introduction

Postoperative hemorrhage (POH) following supratentorial brain tumor resection is a serious complication associated with acute neurological deterioration, reoperation, and increased mortality (1, 2). Although postoperative monitoring in the neurosurgical intensive care unit (NICU) is routine (3, 4), the occurrence of POH can significantly complicate the clinical course (5). Traditionally, the risk of POH has been attributed to static clinical factors such as age, tumor size, preoperative coagulation profiles, perioperative blood pressure, and surgical technique (6–12). However, these predictors are largely derived from general populations, and their utility in the resource-intensive subgroup of patients requiring prolonged NICU care remains uncertain. Furthermore, these factors primarily reflect external or anatomical conditions and often fail to capture the dynamic physiological state of the host that may be associated with postoperative bleeding.

Systemic inflammation is increasingly recognized as a hallmark of cancer biology and an important contributor to dysregulation of the immune–hemostatic balance (13, 14). Inflammatory activation may increase bleeding risk by disrupting vascular endothelial integrity, altering coagulation–fibrinolysis pathways, and impairing platelet function (14, 15). Peripheral blood indices derived from routine blood counts, including the neutrophil-to-lymphocyte ratio (NLR), platelet-to-lymphocyte ratio (PLR), and systemic inflammation response index (SIRI), provide practical surrogates of this systemic inflammatory state (16–18). In related clinical settings, these indices have been associated with hematoma expansion in spontaneous intracerebral hemorrhage (19–23) and with outcomes in patients with brain tumors (24–26), but their association with postoperative hemorrhage after brain tumor resection has not been studied.

In this study, we aimed to evaluate the associations of preoperative NLR, PLR, and SIRI with postoperative hemorrhage in patients requiring prolonged neurosurgical intensive care following elective supratentorial brain tumor resection, and to develop an integrated risk stratification model for early postoperative risk assessment.

## 2 Materials and methods

### 2.1 Study design

This retrospective cohort study was conducted in the 94-bed neurosurgical intensive care unit (NICU) of Huashan Hospital, Fudan University, where postoperative NICU admission is routine after elective supratentorial brain tumor resection. We screened all adult patients admitted to the NICU following such procedures between January 1, 2023, and December 31, 2024. The study protocol adhered to the Declaration of Helsinki and was approved by the Institutional Review Board of Huashan Hospital (No. 2025-648). The study was registered in the Chinese Clinical Trial Registry (ChiCTR2500104426). Given the retrospective design, the requirement for informed consent was waived. To ensure data reliability, two independent investigators (H.Y. and Y.G.), blinded to the study hypothesis and outcome classification, independently extracted data from electronic medical records and assessed all postoperative CT scans. Discrepancies were resolved by consensus or adjudication by a senior clinician (L.W.). This study is reported in accordance with the Strengthening the Reporting of Observational Studies in Epidemiology (STROBE) guidelines.

### 2.2 Study participants

Clinically stable patients are typically transferred from the NICU to a general ward within 48 hours after surgery. In the present analysis, we focused on a resource-intensive subgroup of patients who remained in the NICU for more than 48 hours, defined *a priori* as prolonged NICU stay. Eligible patients met the following criteria: (1) age ≥ 18 years; (2) elective supratentorial brain tumor resection; and (3) postoperative NICU stay > 48 hours. Exclusion criteria were: (1) active preoperative infection; (2) preoperative use of steroids, immunosuppressants, radiotherapy, or chemotherapy; (3) history of hematologic disorders; or (4) missing essential clinical or laboratory data. Patients discharged from the NICU within 48 hours and subsequently readmitted due to postoperative complications were not included. Patients admitted between January 2023 and June 2024 constituted the development cohort, while those admitted between July 2024 and December 2024 formed an independent temporal validation cohort.

### 2.3 Clinical data collection

Data were extracted from electronic medical records, including demographics and comorbidities, prior antiplatelet or anticoagulant use (all agents were discontinued at least one week before surgery per institutional protocol), preoperative functional status assessed by the Karnofsky Performance Status (KPS), and level of consciousness assessed by the Glasgow Coma Scale (GCS). Tumor-related variables included tumor type, anatomical location, maximum tumor diameter on preoperative contrast-enhanced magnetic resonance imaging, and tumor status (primary vs recurrent). Surgical variables comprised surgical approach, extent of resection (classified as gross-total, subtotal, or partial based on the surgeon’s intraoperative assessment as documented in the operative record), operative time, intraoperative blood loss, and intraoperative red blood cell transfusion volume. Postoperative systolic and diastolic blood pressure (SBP/DBP) were defined as the highest measurements recorded between the end of surgery and discharge from the post-anesthesia care unit (PACU). Neurological status (GCS score) and endotracheal intubation status were recorded upon NICU admission. Preoperative laboratory data, including complete blood counts and coagulation profiles, were obtained within 3 days before surgery and prior to any postoperative events. Inflammatory indices were calculated as follows: SIRI = (neutrophil count × monocyte count)/lymphocyte count; NLR = neutrophil count/lymphocyte count; and PLR = platelet count/lymphocyte count.

### 2.4 Postoperative hemorrhage assessment

At our center, all patients routinely undergo non-contrast head computed tomography (NCCT) upon transfer from the PACU, which served as the baseline scan for POH assessment. Subsequent management followed a standardized protocol: (1) patients with hematomas causing significant mass effect requiring surgical evacuation returned to the operating room and were then admitted to the NICU; (2) patients with hematomas without significant mass effect were transferred to the NICU for close monitoring regardless of symptoms; and (3) patients without detectable hemorrhage received routine NICU monitoring. For patients remaining in the NICU, scheduled follow-up NCCT scans were performed within 48 hours postoperatively and again either prior to NICU discharge or on postoperative day 7, whichever occurred first. Additional scans were obtained in cases of neurological deterioration, defined as new focal deficits, decreased level of consciousness, seizures, or signs of elevated intracranial pressure (e.g., severe headache or vomiting).

The primary outcome was any radiographic POH, defined as any new hemorrhage detected on NCCT within 7 days after surgery, irrespective of symptoms or need for intervention. To reflect differing clinical relevance, POH was additionally classified into two secondary categories: clinically significant hemorrhage, defined as radiographic POH accompanied by attributable neurological deterioration (as defined above); and POH requiring surgical reintervention, defined as hemorrhage necessitating a return to the operating room for evacuation. Hemorrhage timing was calculated from the baseline NCCT scan. Hematoma volume was estimated using the ABC/2 formula, where A, B, and C represent the three largest perpendicular diameters on axial CT images. Hemorrhage locations were categorized as resection cavity, epidural, subdural, or remote sites.

Interobserver agreement for the assessment of hemorrhage on the postoperative CT scans, evaluated in a randomly selected subset of 100 cases, was a Cohen’s kappa of 0.85 (95% CI 0.73–0.96) for detection and an intraclass correlation coefficient of 0.95 (95% CI 0.89–0.97) for volume measurement.

### 2.5 Statistical analysis

Continuous variables are presented as mean ± standard deviation (SD) or median with interquartile range (IQR), as appropriate, and categorical variables as frequencies with percentages. Group comparisons were performed using the independent-samples t-test or Mann-Whitney U test for continuous variables and the chi-square test or Fisher’s exact test for categorical variables. Preoperative blood count data were missing for 3.5% (21/608) of clinically eligible patients because the tests had been performed at outside institutions and recorded only on paper; these patients were excluded, and complete-case analysis was performed. Univariable logistic regression was used to identify candidate risk factors for POH. Variables with *P* < 0.05 in univariable analyses, along with pre-specified clinically relevant variables, were entered into multivariable logistic regression models. To avoid multicollinearity, component cell counts of composite inflammatory indices were excluded, and variance inflation factors (VIF > 5) were used to assess remaining collinearity. Backward stepwise selection was applied to identify independent risk factors. Restricted cubic splines (RCS) with four knots were used to assess potential non-linear associations of SIRI and PLR with POH.

The discriminatory performance of inflammatory indices was evaluated using receiver operating characteristic (ROC) curves and area under the curve (AUC), with comparisons performed using DeLong’s test. Incremental discriminative value beyond clinical variables was assessed using integrated discrimination improvement (IDI) and continuous (category-free) net reclassification improvement (NRI). Combined models integrating clinical risk factors with inflammatory indices were compared, and the final model was selected based on the highest AUC and lowest Akaike Information Criterion (AIC). Because the candidate variables included both preoperative and early postoperative measurements, a model restricted to preoperative risk factors was also fitted for comparison. To evaluate robustness, sensitivity analyses were performed: (1) exclusion of outliers for each inflammatory index using the IQR method (defined as values below Q1 – 1.5 × IQR or above Q3 + 1.5 × IQR); (2) exclusion of patients with hemorrhage on baseline postoperative NCCT; (3) further adjustment of the final model for perioperative variables (extent of resection, surgical approach, operative time, intraoperative blood loss, and intraoperative transfusion); and (4) testing for interactions between inflammatory indices and significant clinical risk factors. Internal validation was performed using 1,000 bootstrap resamples to estimate the optimism-corrected C-index, calibration slope, and intercept. The final model was subsequently validated in the temporal validation cohort, with performance assessed by discrimination (AUC), calibration (calibration slope, intercept, Brier score, and Hosmer–Lemeshow test), and clinical utility (decision curve analysis). Exploratory analyses examined associations between the final-model variables and the more severe outcome tiers (clinically significant POH and POH requiring surgical intervention), as well as effect modification by tumor type (high-grade glioma vs others). All analyses were performed using R software (version 4.5.1), and a two-sided *P* value < 0.05 was considered statistically significant.

## 3 Results

### 3.1 Baseline characteristics

**Figure 1:**
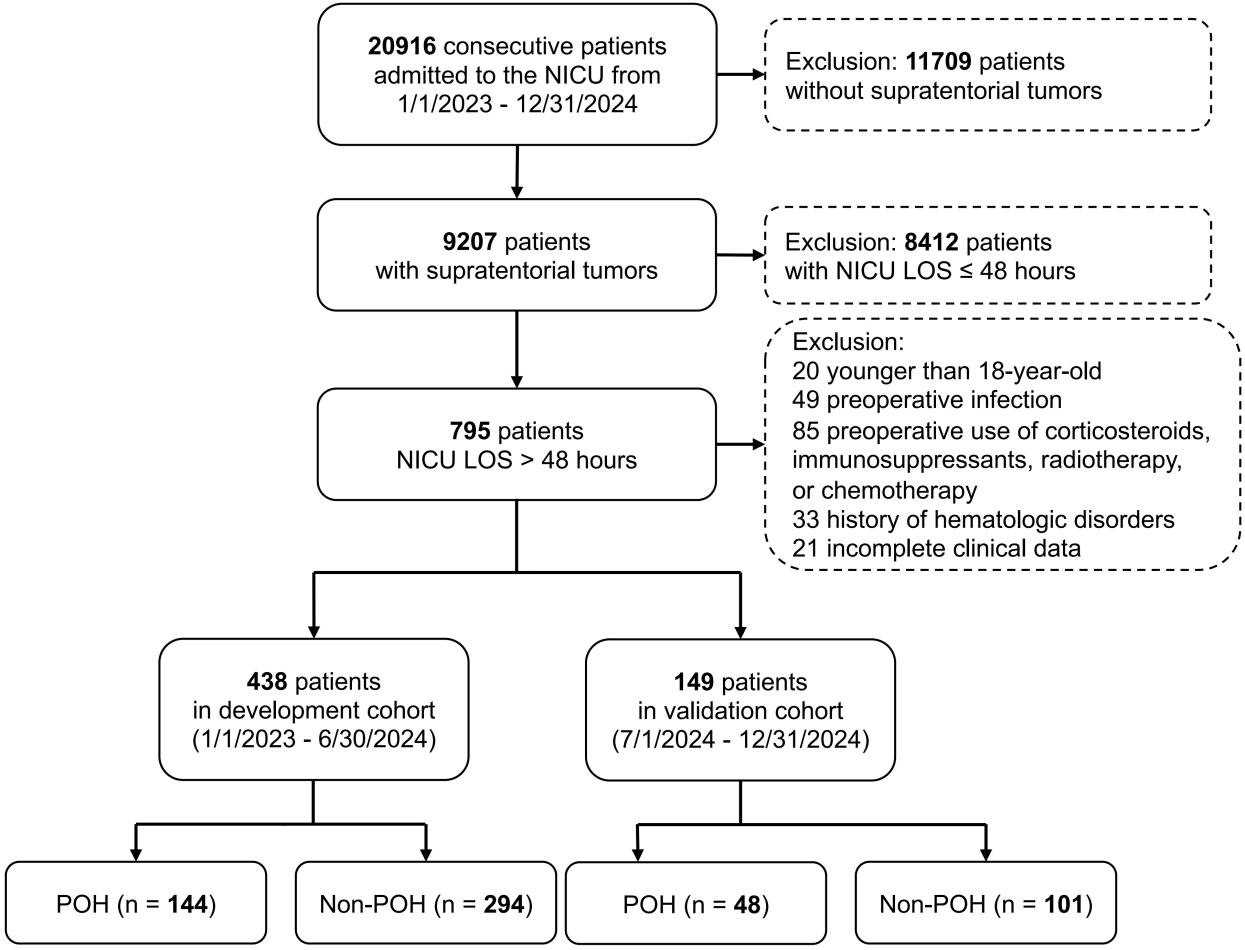
Flow chart of patient recruitment. NICU, neurosurgical intensive care unit; LOS, length of stay; POH, postoperative hemorrhage; Non-POH, Non-postoperative hemorrhage.

A total of 587 adult patients with a postoperative NICU stay exceeding 48 hours after elective supratentorial tumor resection were included (**Figure 1**), comprising 438 patients in the development cohort and 149 in the validation cohort. Baseline characteristics were comparable between the two cohorts (**Table 1**). In the development cohort, the median age was 60.0 years (IQR 50.0–67.0), and 50.7% of patients were male; corresponding values in the validation cohort were 61.0 years (IQR 53.0–69.0) and 55.0% male. All patients underwent elective surgery, and 93.0% (546/587) had a preoperative GCS score of 15. At NICU admission, 72.4% (425/587) had a GCS ≤ 8 and 80.6% (473/587) required endotracheal intubation. Any radiographic POH occurred in 32.9% (144/438) of patients in the development cohort and 32.2% (48/149) in the validation cohort. Within these, 102 and 36 patients had clinically significant hemorrhage, of whom 37 and 11 required surgical reinterventions, respectively (**Supplementary Table 1**). Hemorrhage was already evident on the baseline CT in 38 (26.4%) and 11 (22.9%) of these patients, with the remainder being delayed or *de novo*; most events were detected within 48 hours of surgery (91.0% and 87.5%, respectively). Patients with POH had a longer NICU stay than those without (7.0 [IQR 4.0–11.0] vs 6.0 [IQR 4.0–8.0] days; *P* < 0.001).

**Table 1:** Baseline characteristics of the development and validation cohort.

Variables	Development cohort (n = 438)	Validation cohort (n = 149)	*P* value
Demographics			
Age, years	60.0 (50.0, 67.0)	61.0 (53.0, 69.0)	0.135
Gender, male	222 (50.7%)	82 (55%)	0.411
Clinical characteristics			
Hypertension	210 (47.9%)	83 (55.7%)	0.123
Diabetes	118 (26.9%)	49 (32.9%)	0.199
Prior antiplatelet/anticoagulant use	7 (1.6%)	4 (2.7%)	0.483
Preoperative KPS	90.0 (80.0, 90.0)	90.0 (80.0, 90.0)	0.983
Preoperative GCS	15.0 (15.0, 15.0)	15.0 (15.0, 15.0)	0.564
Preoperative GCS = 15	409 (93.4%)	137 (91.9%)	0.684
Postoperative Admission Status			
GCS at admission	4.0 (3.0, 10.0)	4.0 (3.0, 8.0)	0.970
GCS at admission ≤ 8	312 (71.2%)	113 (75.8%)	0.327
Endotracheal tube at admission	348 (79.5%)	125 (83.9%)	0.287
Tumor characteristics			
Tumor type			0.380
High-grade glioma	211 (48.2%)	87 (58.4%)	
Meningioma	108 (24.7%)	29 (19.5%)	
Pituitary adenoma	40 (9.1%)	9 (6%)	
Metastasis	29 (6.6%)	10 (6.7%)	
Low-grade glioma	27 (6.2%)	8 (5.4%)	
Others	23 (5.3%)	6 (4%)	
Tumor site			0.482
Lobe	220 (50.2%)	85 (57%)	
Skull base	94 (21.5%)	23 (15.4%)	
Basal ganglia	96 (21.9%)	34 (22.8%)	
Ventricle	22 (5%)	6 (4%)	
Pineal region	6 (1.4%)	1 (0.7%)	
Tumor diameter, cm	5.0 (3.6, 6.0)	4.9 (4.1, 6.0)	0.321
Tumor status			0.997
Primary	386 (88.1%)	132 (88.6%)	
Recurrent	52 (11.9%)	17 (11.4%)	
Surgical details			
Surgical approach			0.139
Craniotomy	356 (81.3%)	130 (87.2%)	
Endoscopic surgery	47 (10.7%)	8 (5.4%)	
Keyhole surgery	35 (8%)	11 (7.4%)	
Operative time, hours	5.0 (4.0, 7.0)	5.0 (4.0, 6.0)	0.278
Intraoperative blood loss, ml	400.0 (300.0, 500.0)	400.0 (300.0, 600.0)	0.072
Intraoperative RBC transfusion			1.000
≤ 400 ml	392 (89.5%)	134 (89.9%)	
> 400 ml	46 (10.5%)	15 (10.1%)	
Extent of resection			0.339
Gross total	293 (66.9%)	98 (65.8%)	
Subtotal	92 (21%)	38 (25.5%)	
Partial	53 (12.1%)	13 (8.7%)	
Postoperative blood pressure			
Systolic blood pressure (mmHg)	139.0 (126.0, 150.0)	137.0 (126.0, 146.0)	0.114
Diastolic blood pressure (mmHg)	82.0 (74.0, 90.0)	81.0 (72.0, 89.0)	0.521
Laboratory data			
White blood cells, ×109/L	6.3 (5.0, 7.7)	6.5 (5.3, 7.9)	0.278
Red blood cells, ×1012/L	4.4 (4.1, 4.7)	4.5 (4.2, 4.7)	0.173
Hemoglobin, g/L	131.0 (123.0, 141.0)	133.0 (123.0, 141.0)	0.628
Platelet, ×109/L	195.0 (161.0, 240.0)	207.0 (164.0, 245.0)	0.153
Neutrophils, ×109/L	4.1 (3.0, 5.2)	4.2 (3.1, 5.5)	0.442
Lymphocytes, ×109/L	1.5 (1.1, 2.0)	1.5 (1.1, 2.0)	0.799
Monocytes, ×109/L	0.4 (0.3, 0.5)	0.4 (0.3, 0.5)	0.998
Prothrombin time, s	11.3 (10.8, 11.7)	11.2 (10.8, 11.6)	0.663
International normalized ratio	1.0 (0.9, 1.0)	1.0 (0.9, 1.0)	0.712
Activated partial thromboplastin time, s	26.9 (25.2, 28.2)	26.4 (25.4, 27.8)	0.213
SIRI	1.1 (0.7, 1.8)	1.1 (0.7, 1.6)	0.941
NLR	2.7 (1.9, 3.8)	2.6 (1.8, 4.2)	0.954
PLR	126.3 (98.6, 173.5)	130.3 (103.8, 176.9)	0.561

Data are presented as n (%) or median (IQR). KPS, Karnofsky Performance Status; GCS, Glasgow Coma Scale; RBC, red blood cells; SIRI, systemic inflammation response index; NLR, neutrophil-to-lymphocyte ratio; PLR, platelet-to-lymphocyte ratio.

### 3.2 Associations between inflammatory indices and POH

**Figure 2:**
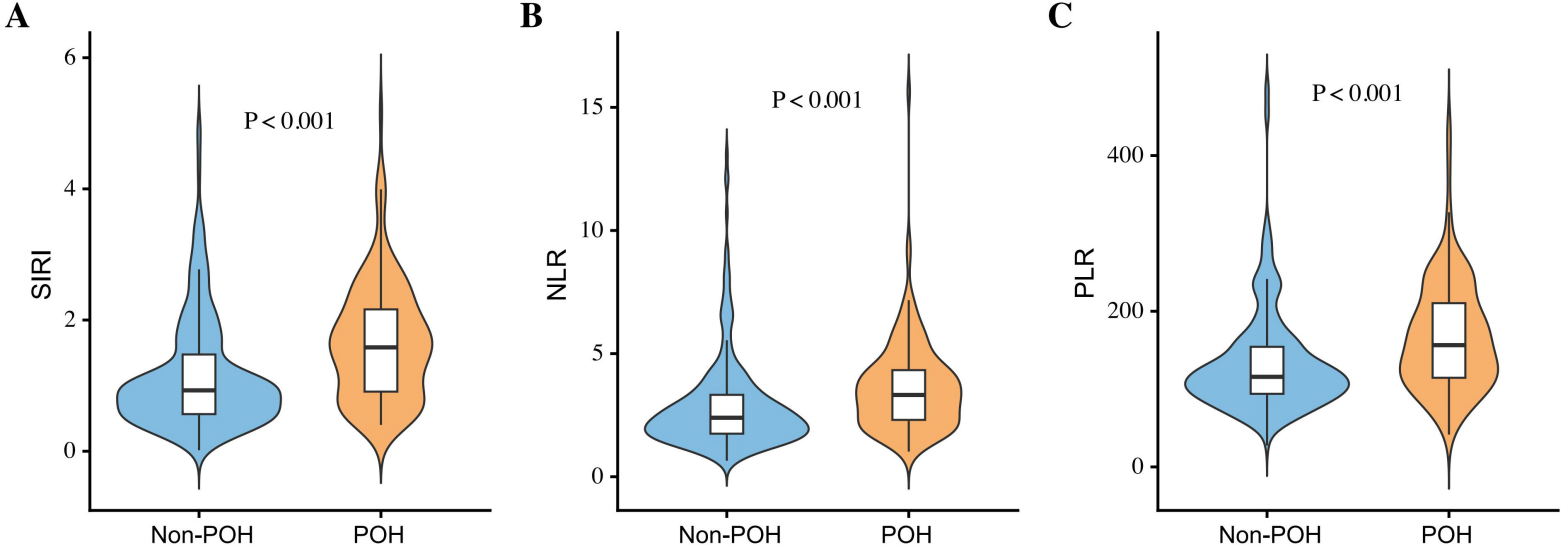
Distribution of preoperative inflammatory indices. Boxplots compare **(A)** SIRI, **(B)** NLR, and **(C)** PLR between Non-POH (n=294) and POH (n=144) groups. All three indices were significantly higher in the POH group (*P* < 0.001). SIRI, systemic inflammation response index; NLR, neutrophil-to-lymphocyte ratio; PLR, platelet-to-lymphocyte ratio; POH, postoperative hemorrhage; Non-POH, Non-postoperative hemorrhage.

Comparisons between patients with and without POH in the development cohort are shown in **Table 2**. Preoperative SIRI, NLR, and PLR were all higher in patients who developed POH (all *P* < 0.001; **Figure 2**). Variables associated with POH on univariable analysis are presented in **Table 3**. After excluding the component blood-cell counts to avoid collinearity and confirming that all VIFs < 5 (**Supplementary Table 2**), multivariable analysis identified SIRI (adjusted OR [aOR] 1.56, 95% CI 1.18-2.06; *P* = 0.002) and PLR (aOR 1.49 per 100-unit increase, 95% CI 1.05-2.11; *P* = 0.025) as independently associated with POH, together with basal ganglia location (aOR 1.88; *P* = 0.016), tumor diameter (aOR 1.28; *P* < 0.001), and postoperative SBP (aOR 1.03; *P* < 0.001). NLR and extent of resection (subtotal vs gross-total, OR 1.83; *P* = 0.014) were each associated with POH on univariable analysis but were not independent after multivariable adjustment; the SIRI and PLR associations were unchanged when extent of resection was included. RCS analysis showed that the associations of SIRI and PLR with POH were monotonic but non-linear, attenuating at higher values (non-linearity *P* < 0.001 and *P* = 0.003, respectively; **Supplementary Figure 1**).

**Table 2:** Comparison of patients in the development cohort.

Variables	POH (n = 144)	Non-POH (n = 294)	*P* value
Demographics			
Age, years	61.5 (53.0, 68.0)	59.0 (48.0, 66.0)	0.034
Gender, male	75 (52.1%)	147 (50%)	0.758
Clinical characteristics			
Hypertension	62 (43.1%)	148 (50.3%)	0.183
Diabetes	43 (29.9%)	75 (25.5%)	0.396
Preoperative KPS	90.0 (80.0, 90.0)	90.0 (80.0, 90.0)	0.181
Prior antiplatelet/anticoagulant use	3 (2.1%)	4 (1.4%)	0.689
Tumor characteristics			
Tumor type			0.232
High-grade glioma	68 (47.2%)	143 (48.6%)	
Meningioma	42 (29.2%)	66 (22.4%)	
Pituitary adenoma	11 (7.6%)	29 (9.9%)	
Metastasis	5 (3.5%)	24 (8.2%)	
Low-grade glioma	8 (5.6%)	19 (6.5%)	
Others	10 (6.9%)	13 (4.4%)	
Tumor site			0.070
Lobe	64 (44.4%)	156 (53.1%)	
Skull base	27 (18.8%)	67 (22.8%)	
Basal ganglia	43 (29.9%)	53 (18%)	
Ventricle	8 (5.6%)	14 (4.8%)	
Pineal region	2 (1.4%)	4 (1.4%)	
Tumor diameter, cm	5.3 (4.1, 6.5)	4.6 (3.2, 5.8)	<0.001
Tumor status			0.414
Primary	130 (90.3%)	256 (87.1%)	
Recurrent	14 (9.7%)	38 (12.9%)	
Surgical details			
Surgical approach			0.323
Craniotomy	122 (84.7%)	234 (79.6%)	
Endoscopic surgery	11 (7.6%)	36 (12.2%)	
Keyhole surgery	11 (7.6%)	24 (8.2%)	
Operative time, hours	5.5 (4.0, 7.0)	5.0 (4.0, 6.5)	0.268
Intraoperative blood loss, ml	400.0 (300.0, 600.0)	400.0 (300.0, 500.0)	0.142
Intraoperative RBC transfusion			0.263
≤ 400 ml	125 (86.8%)	267 (90.8%)	
> 400 ml	19 (13.2%)	27 (9.2%)	
Extent of resection			0.027
Gross total	84 (58.3%)	209 (71.1%)	
Subtotal	39 (27.1%)	53 (18.0%)	
Partial	21 (14.6%)	32 (10.9%)	
Postoperative blood pressure			
Systolic blood pressure (mmHg)	145.2 ± 19.8	135.5 ± 18.0	<0.001
Diastolic blood pressure (mmHg)	85.0 ± 12.4	80.8 ± 11.5	<0.001
Laboratory data			
White blood cells, ×109/L	6.0 (4.9, 7.4)	6.5 (5.0, 7.8)	0.096
Red blood cells, ×1012/L	4.4 (4.1, 4.7)	4.5 (4.1, 4.8)	0.449
Hemoglobin, g/L	131.0 (124.0, 140.0)	131.0 (123.0, 141.0)	0.794
Platelet, ×109/L	189.0 (159.5, 234.0)	195.5 (162.0, 241.0)	0.353
Neutrophils, ×109/L	4.1 (3.0, 5.3)	4.1 (3.0, 5.2)	0.906
Lymphocytes, ×109/L	1.3 (1.0, 1.6)	1.7 (1.3, 2.1)	<0.001
Monocytes, ×109/L	0.5 (0.4, 0.5)	0.4 (0.3, 0.5)	<0.001
Prothrombin time, s	11.3 (10.9, 11.8)	11.2 (10.8, 11.7)	0.283
International normalized ratio	1.0 (0.9, 1.0)	1.0 (0.9, 1.0)	0.265
Activated partial thromboplastin time, s	26.9 (25.4, 28.6)	26.8 (25.2, 28.2)	0.534
SIRI	1.6 (0.9, 2.2)	0.9 (0.6, 1.5)	<0.001
NLR	3.3 (2.3, 4.4)	2.4 (1.8, 3.3)	<0.001
PLR	156.4 (114.4, 210.8)	115.7 (93.8, 154.6)	<0.001

Data are presented as n (%), mean ± SD, or median (IQR). POH, postoperative hemorrhage; KPS, Karnofsky Performance Status; RBC, red blood cells; SIRI, systemic inflammation response index; NLR, neutrophil-to-lymphocyte ratio; PLR, platelet-to-lymphocyte ratio.

**Table 3:** Univariable and multivariable analyses for postoperative hemorrhage in the development cohort.

Variables	Univariable analysis		Multivariable analysis	
	OR (95% CI)	*P* value	OR (95% CI)	*P* value
Age, years	1.02 (1.00–1.03)	0.045		
Basal ganglia location (vs other sites)	1.94 (1.22–3.08)	0.005	1.88 (1.12–3.15)	0.016
Tumor diameter, cm	1.38 (1.21–1.57)	<0.001	1.28 (1.11–1.47)	<0.001
Extent of resection				
Gross total	Reference			
Subtotal	1.83 (1.13–2.97)	0.014		
Partial	1.63 (0.89–2.99)	0.113		
Postoperative SBP, mmHg	1.03 (1.02–1.04)	<0.001	1.03 (1.02–1.04)	<0.001
Postoperative DBP, mmHg	1.03 (1.01–1.05)	<0.001		
SIRI, per 1 unit	1.86 (1.47–2.35)	<0.001	1.56 (1.18–2.06)	0.002
NLR, per 1 unit	1.24 (1.11–1.38)	<0.001		
PLR, per 100 units	2.00 (1.48–2.71)	<0.001	1.49 (1.05–2.11)	0.025

OR, odds ratio; CI, confidence interval; SBP, systolic blood pressure; DBP, diastolic blood pressure; SIRI, systemic inflammation response index; NLR, neutrophil-to-lymphocyte ratio; PLR, platelet-to-lymphocyte ratio.

### 3.3 Discrimination and model development

**Figure 3:**
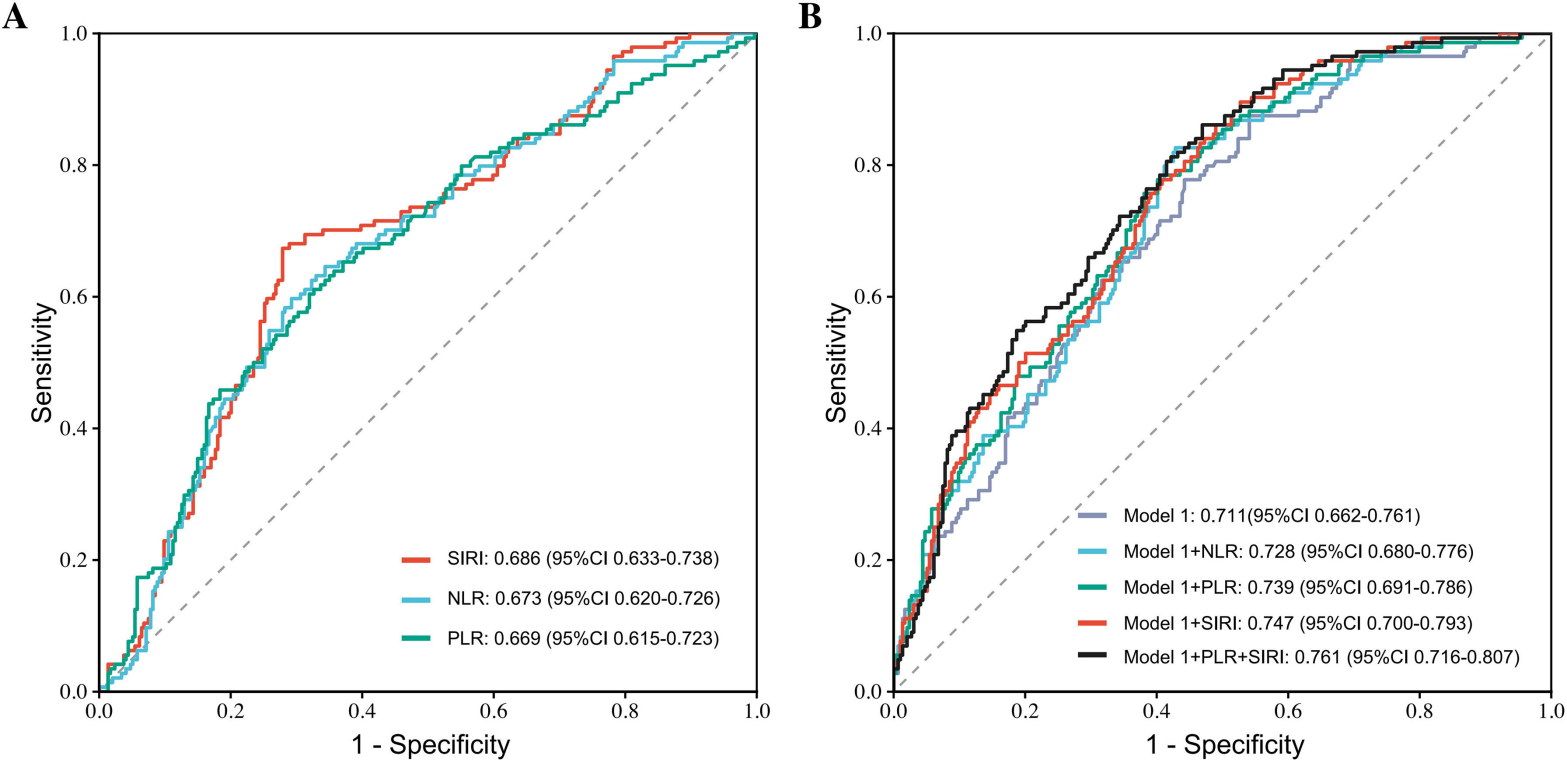
Discrimination of inflammatory indices and combined models for postoperative hemorrhage. **(A)** Receiver operating characteristic (ROC) curves comparing SIRI, NLR, and PLR. **(B)** ROC curves comparing the base clinical model (Model 1) with models additionally incorporating NLR, PLR, SIRI, or both PLR and SIRI. SIRI, systemic inflammation response index; NLR, neutrophil-to-lymphocyte ratio; PLR, platelet-to-lymphocyte ratio; AUC, area under the ROC curve; CI, confidence interval. Model 1: basal ganglia location, tumor diameter, and postoperative systolic blood pressure.

The discriminatory performance of individual inflammatory indices was modest (SIRI AUC 0.686, NLR 0.673, PLR 0.669), with no statistically significant differences (DeLong’s test, *P* > 0.05; **Figure 3A**; **Supplementary Table 3**). When added separately to the base clinical model (Model 1: basal ganglia location, tumor diameter, and postoperative SBP), SIRI yielded the largest improvement in discrimination (IDI 0.043, *P* < 0.001) and was the only index to significantly improve the reclassification of POH events (event NRI 0.208, *P* = 0.011; **Supplementary Table 4**). The final model combined SIRI and PLR with the clinical risk factors (Model 1 + SIRI + PLR), achieving an AUC 0.761 (95% CI 0.716-0.807) and the lowest AIC (484.13) among the models compared (**Figure 3B**; **Table 4**). A preoperative-only model excluding postoperative SBP was also evaluated; it yielded an AUC of 0.727 (95% CI 0.679–0.776) with a higher AIC (509.91; **Table 4**). The difference in AUC did not reach statistical significance (DeLong’s test, *P* = 0.052), while the AIC was 25.78 lower in the final model. The fitted equation was:

**Table 4:** Comparison of models for postoperative hemorrhage.

Models	AUC (95% CI)	AIC	*P* value^Δ^
Model 1	0.711 (0.662-0.761)	506.25	0.002
Model 1 + NLR	0.728 (0.680-0.776)	497.57	< 0.001
Model 1 + PLR	0.739 (0.691-0.786)	492.30	0.020
Model 1 + SIRI	0.747 (0.700-0.793)	487.29	0.047
Model 1 + PLR + SIRI	0.761 (0.716-0.807)	484.13	/
Preoperative-only model*	0.727 (0.679-0.776)	509.91	0.052

AUC, area under receiver operating characteristic; CI, confidence interval; AIC, Akaike Information Criterion; NLR, neutrophil-to-lymphocyte ratio; PLR, platelet-to-lymphocyte ratio; SIRI, systemic inflammation response index.

Model 1: basal ganglia location, tumor diameter, postoperative systolic blood pressure.

*Preoperative-only model: basal ganglia location, tumor diameter, SIRI, and PLR.

^Δ^ DeLong's test for AUC of each model vs Model 1 + PLR + SIRI

Logit (*P*) = −7.72 + 0.63 × Basal ganglia location + 0.24 × Tumor diameter + 0.032 × Postoperative SBP + 0.44 × SIRI + 0.40 × (PLR/100) (**Supplementary Table 5**).

### 3.4 Temporal validation

**Figure 4:**
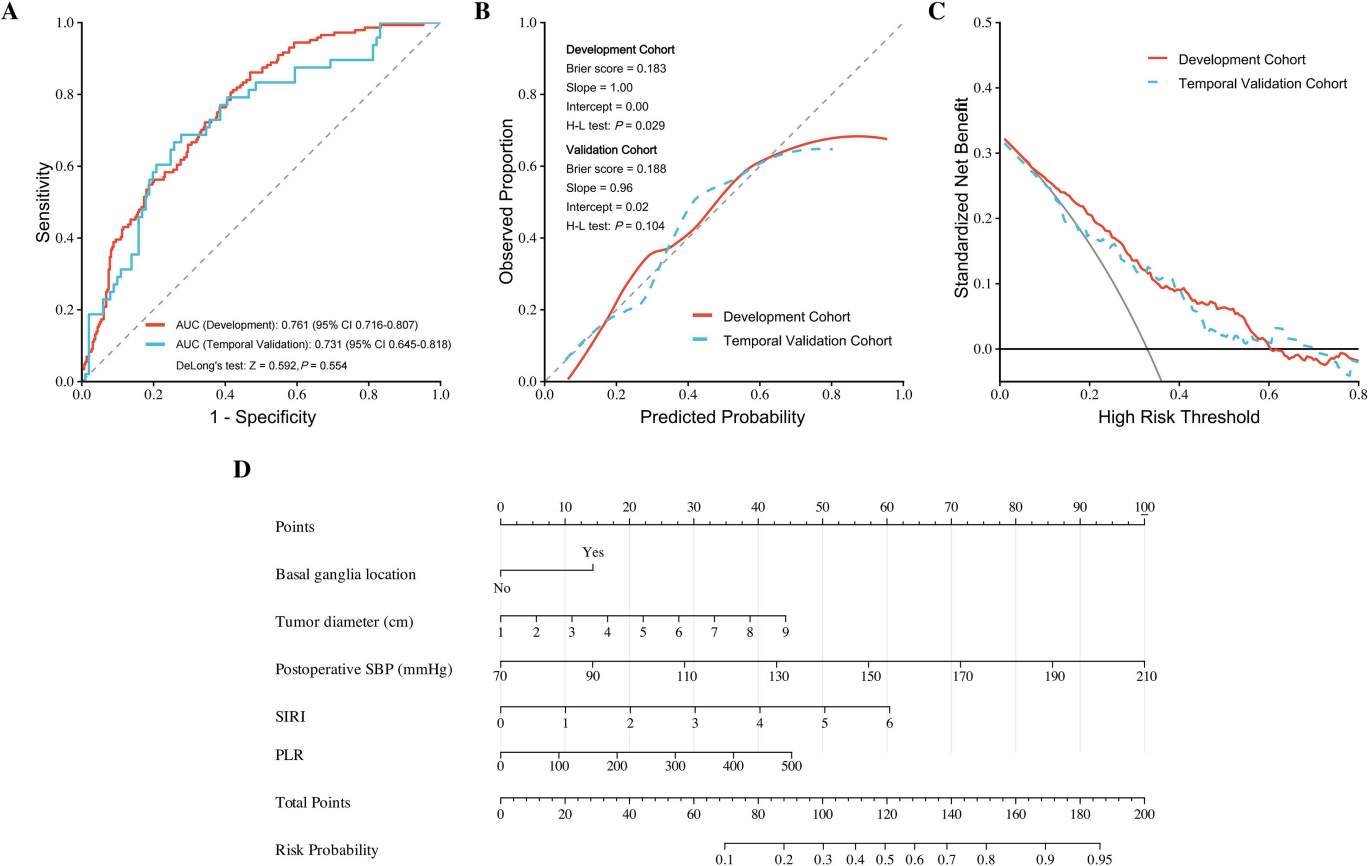
Temporal validation and clinical application of the final early postoperative risk stratification model. **(A)** Receiver operating characteristic (ROC) curves of the final model in the development and temporal validation cohorts. **(B)** Calibration plots for the development and temporal validation cohorts. **(C)** Decision curve analysis showing the net benefit across threshold probabilities in both cohorts. **(D)** Nomogram derived from the development cohort for estimating individual postoperative hemorrhage risk at NICU admission. AUC, area under the ROC curve; CI, confidence interval; H-L test, Hosmer-Lemeshow test; SBP, systolic blood pressure; SIRI, systemic inflammation response index; PLR, platelet-to-lymphocyte ratio.

Internal validation with 1,000 bootstrap resamples yielded an optimism-corrected C-index of 0.751, a calibration slope of 0.94, and an intercept of −0.04, indicating modest optimism in the apparent estimates. In the temporal validation cohort, the final model yielded an AUC of 0.731 (95% CI 0.645-0.818), with no significant difference from the development cohort (DeLong’s test, Z = 0.592, *P* = 0.554; **Figure 4A**). Calibration analysis showed a calibration slope of 0.96 and an intercept of 0.02 in the validation cohort, with a Brier score of 0.188 (Hosmer-Lemeshow test, *P* = 0.104; **Figure 4B**). Decision curve analysis indicated that the model provided a net benefit exceeding the treat-all strategy across threshold probabilities of approximately 0.2 to 0.6 in both cohorts (**Figure 4C**). A nomogram was constructed to facilitate clinical application of this risk stratification tool (**Figure 4D**).

### 3.5 Sensitivity and robustness analyses

To assess the robustness of the associations of SIRI and PLR with POH, sensitivity analyses were performed by sequentially excluding outliers of each inflammatory index identified by the boxplot method (**Supplementary Figure 2**). The aOR of SIRI (range 1.69–2.44) and PLR (per 100 units, range 1.71–2.91) remained statistically significant and consistent in direction across all subsets, whereas NLR showed no independent association (**Supplementary Table 6**). The associations also persisted after excluding patients with hemorrhage identified on the baseline postoperative CT (n = 400; SIRI aOR 1.48, *P* = 0.011; PLR aOR 1.69, *P* = 0.005), supporting an association with delayed rather than immediate hemorrhage. Further adjustment of the final model for perioperative variables left the SIRI and PLR estimates unchanged (aOR 1.54 and 1.52 per 100 units, respectively; **Supplementary Table 7**), and none of these surgical variables was independently associated with POH. Calibration of the development cohort, which was influenced by a small number of extreme inflammatory values in the primary analysis, also improved after outlier exclusion (**Supplementary Figure 3**). No significant interaction was observed between the inflammatory indices and clinical risk factors (all interaction *P* > 0.05; **Supplementary Table 8**).

### 3.6 Exploratory analyses

Two sets of exploratory analyses were performed. Against the more severe outcome tiers, PLR was associated with clinically significant POH (n = 102; aOR 1.45 per 100 units, 95% CI 1.01–2.07, *P* = 0.043), while SIRI attenuated to borderline significance (aOR 1.29, *P* = 0.082); for POH requiring surgical reintervention (n = 37), neither index stayed independent and postoperative SBP was the dominant correlate (**Supplementary Table 9**). Effect modification by tumor type (high-grade glioma vs other histologies) suggested possible interactions with both SIRI (OR 0.58, 95% CI 0.34–1.00, *P* = 0.049) and PLR (OR 0.45, 95% CI 0.22–0.89, *P* = 0.022; **Supplementary Table 8**).

## 4 Discussion

POH is a severe complication following supratentorial brain tumor resection, associated with increased morbidity and mortality (1, 5). In this study, we specifically focused on a high-risk and resource-intensive subgroup of patients requiring prolonged postoperative NICU care (> 48 hours), a population in whom early identification of hemorrhagic risk is clinically critical yet insufficiently characterized in existing literature (27–29). Our findings suggest that preoperative systemic inflammatory status, captured by SIRI and PLR, but not NLR, is independently associated with POH. Among these indices, SIRI showed the most consistent relationship and provided the greatest incremental discriminative value beyond established clinical risk factors. Together, these results suggest that postoperative hemorrhagic risk in this population may be influenced not only by surgical and anatomical factors, but also by the preoperative systemic inflammatory state of the host.

Systemic inflammation is known to influence both tumor biology and vascular stability through its effects on endothelial function, coagulation pathways, and immune regulation (13–15). In this study, however, not all inflammation-related indices showed equivalent relevance to POH risk. Although NLR is widely used as a general marker of systemic inflammation and physiological stress (19, 23–25), it was not an independent risk factor for POH after multivariable adjustment. In contrast, SIRI and PLR remained independently associated with POH, suggesting that inflammatory indices incorporating monocyte- and platelet-related components may better reflect the biological processes related to postoperative bleeding. When added individually to a base clinical model, the incremental discriminative contribution followed the order SIRI > PLR > NLR, with only SIRI significantly improving reclassification of POH events. These findings indicate that composite inflammatory indices capturing multiple immune–hematologic pathways may be more informative than simpler leukocyte ratios in identifying hemorrhage-prone patients.

SIRI integrates neutrophil, monocyte, and lymphocyte counts, thereby capturing a composite inflammatory and immune-regulatory state that is not reflected by simpler leukocyte ratios (24, 25). Neutrophils can contribute to vascular injury through the release of matrix metalloproteinases, including MMP-9, which degrade the vascular basement membrane and increase permeability (30). Monocytes, as precursors of tumor-associated macrophages (TAMs), promote aberrant angiogenesis and structural immaturity of tumor-associated vessels, leading to increased vascular fragility (31, 32). Concurrent lymphopenia may further impair immune-mediated vascular repair mechanisms (33). In combination, these alterations may render peritumoral microvasculature more susceptible to hemorrhage following surgical stress. PLR reflects a related but distinct pathway linking systemic inflammation to platelet-mediated hemostasis (19). Chronic inflammatory activation can induce abnormal platelet activation, consumption, and functional exhaustion, potentially compromising primary hemostasis during the postoperative period (34, 35). By contrast, NLR incorporates only neutrophil and lymphocyte components and does not account for monocyte-driven vascular remodeling or platelet-related hemostatic imbalance. This biological limitation may explain why NLR was not retained as an independent risk factor for POH in multivariable analyses, despite its widespread use as a general inflammatory marker.

The integrated early postoperative model developed in this study highlights the complementary roles of host inflammatory status and established clinical risk factors in characterizing POH risk. Basal ganglia location, larger tumor diameter, and elevated postoperative SBP are well-recognized contributors to postoperative hemorrhage, reflecting anatomical complexity, surgical extent, and postoperative hemodynamic stress (8, 9, 11, 36, 37). By incorporating preoperative inflammatory indices, the model extends beyond these relatively static factors to capture a systemic inflammatory component of hemorrhagic risk. As postoperative SBP is the only postoperative variable and is recorded during PACU stay, all model inputs become available at NICU admission, enabling risk stratification at the earliest clinically actionable time point. Notably, no significant interactions were observed between SIRI or PLR and major clinical risk factors, indicating that the associations of these inflammatory indices with POH are consistent across clinical contexts and supporting their robustness within the integrated model. Importantly, SIRI and PLR remained independently linked to POH after adjustment for available perioperative and surgical factors, as well as in sensitivity analyses excluding patients with hemorrhage detected on immediate postoperative baseline imaging, a group in whom bleeding is more likely attributable to intraoperative technical factors. This finding suggests that preoperative systemic inflammation may be more closely related to delayed postoperative hemorrhage rather than immediate surgical bleeding. In addition, model calibration in the development cohort was influenced by extreme inflammatory values; exclusion of these outliers improved calibration without materially altering effect estimates (**Supplementary Figure 3**), consistent with the stable calibration observed in the temporal validation cohort. Decision curve analysis showed net benefit across threshold probabilities of approximately 0.2 to 0.6, supporting use of the model to flag higher-risk patients for escalated care, including tighter blood pressure control, earlier surveillance imaging, and more intensive NICU monitoring, although the specific threshold and protocols require prospective validation.

The inflammatory milieu and vascular biology vary substantially across intracranial tumor types, which may influence the development of POH (38, 39). Although tumor histology did not differ significantly between patients with and without POH in the primary analysis (*P* = 0.232), extent of resection was significant on univariable analysis, with greater residual tumor associated with higher POH risk, but lost independence after multivariable adjustment, likely because the anatomical factors that limit complete resection are already captured by tumor diameter and basal ganglia location in the model. Exploratory analyses suggested potential effect modification by tumor type. Specifically, interactions between tumor type (high-grade glioma vs others) and both SIRI and PLR were observed, though the SIRI interaction was borderline (*P* = 0.049), suggesting that the relationship between systemic inflammation and POH risk may differ across tumor subtypes. In the subgroup of patients with severe POH requiring surgical reintervention (n = 37), neither SIRI nor PLR remained independently linked to the outcome after multivariable adjustment, whereas postoperative SBP was the dominant factor (*P* = 0.003), suggesting that hemodynamic rather than inflammatory factors may predominate in more severe hemorrhagic events. These exploratory analyses involved multiple comparisons with limited sample sizes; given the wide confidence intervals observed, the results should be interpreted as hypothesis generating rather than definitive, underscoring the need for validation in larger, histology-stratified cohorts.

This study has several limitations. First, this was a retrospective, single-center analysis, which may limit generalizability. Although temporal validation was performed, prospective multicenter studies are needed to further confirm the robustness and clinical applicability of the proposed model. Second, the requirement for NICU stay exceeding 48 hours may itself be influenced by hemorrhage, although the majority of patients required prolonged care primarily because of postoperative clinical severity (over 70% with GCS ≤ 8 and approximately 80% intubated at NICU admission). The findings should be regarded as applicable to patients requiring prolonged neurosurgical intensive care rather than to the general surgical population; model performance in lower-risk populations remains uncertain. Third, inflammatory indices were assessed using a single preoperative blood sample; perioperative dynamics of systemic inflammation could not be evaluated, and these markers may have been influenced by unmeasured factors such as tumor necrosis or perioperative stress. Fourth, although the final model was further adjusted for available perioperative variables, several potential confounders, including intraoperative blood pressure variability, hemostatic techniques, and postoperative coagulation management, were not systematically recorded, and prior antiplatelet or anticoagulant exposure was rare (n = 7, 1.6%), precluding meaningful adjustment; the possibility of residual confounding from these unmeasured factors should be considered when interpreting the results. Fifth, the primary outcome was any radiographic hemorrhage regardless of clinical significance. Exploratory analyses of the more severe tiers showed that the inflammatory associations attenuated as severity increased, with PLR showing a borderline association with clinically significant POH while SIRI did not, and neither index retained significance for POH requiring surgical reintervention, where the small number of events limits any firm conclusion. Finally, the overall discriminative performance of the model was moderate, and the incremental value contributed by the inflammatory indices, though significant, was modest and should be interpreted with caution; incorporation of additional biological, radiological, or molecular markers may further enhance discrimination.

## 5 Conclusion

This study identified preoperative SIRI and PLR as independent risk factors for POH in patients requiring prolonged NICU stay after supratentorial tumor resection. The early postoperative model integrating SIRI and PLR with basal ganglia location, tumor diameter, and postoperative SBP showed moderate discriminative ability, acceptable calibration, and potential clinical utility. These findings suggest the preoperative systemic inflammatory state as a measurable contributor to POH risk and warrant prospective multicenter validation before broader application.

## Data Availability

The original contributions presented in the study are included in the article/**Supplementary Material**. Further inquiries can be directed to the corresponding author.
