# Supplementary material for: Preoperative systemic inflammatory indices as risk factors for hemorrhage after supratentorial brain tumor resection in patients requiring prolonged neurosurgical intensive care: development and temporal validation of an early postoperative risk stratification model
Source: Front Oncol. 2026 Jul 20;16:1795177. doi: 10.3389/fonc.2026.1795177 (PMC13429410; doi:10.3389/fonc.2026.1795177)
Supplement: Supplementary file 1 [file DataSheet1.docx]

# *Supplementary Material*

# Supplementary Tables

| **Table S1 Clinical features of postoperative hemorrhage in the development and validation cohorts** | | |
| --- | --- | --- |
| **Outcome/features** | **Development cohort (n = 438)** | **Validation cohort (n = 149)** |
| Postoperative hemorrhage (any radiographic) | 144/438 (32.9%) | 48/149 (32.2%) |
| Clinically significant hemorrhage | 102/144 (70.8%) | 36/48 (75.0%) |
| Requiring surgical reintervention | 37/102 (36.3%) | 11/36 (30.6%) |
| Among patients with postoperative hemorrhage | | |
| Timing of hemorrhage detection |  |  |
| Immediate on baseline NCCT | 38 (26.4%) | 11 (22.9%) |
| ≤ 48 hours | 93 (64.6%) | 31 (64.6%) |
| > 48 hours | 13 (9.0%) | 6 (12.5%) |
| Time to detection, hours | 14 (0^*^, 18.5) | 15 (3, 23.2) |
| Hemorrhage volume, ml | 7.8 (5, 18) | 9.2 (6.4, 18.9) |
| Hemorrhage sites |  |  |
| Tumor cavity | 128 (88.8%) | 42 (87.5%) |
| Epidural space | 5 (3.5%) | 2 (4.2%) |
| Subdural space | 6 (4.2%) | 4 (8.3%) |
| Remote location | 5 (3.5%) | 0 (0%) |
| Data are presented as n (%) or median (IQR). For detection timing, time to detection, volume, and site, percentages are relative to all patients with postoperative hemorrhage. NCCT, non-contrast head computed tomography.  *Hemorrhage present on baseline NCCT | | |

| **Table S2** **Variance inflation factors of the variables in the multivariable analysis** | |
| --- | --- |
| **Variables** | **Variance inflation factors** |
| Age | 1.11 |
| Basal ganglia location | 1.13 |
| Tumor diameter | 1.12 |
| Extent of resection* | 1.03 |
| Postoperative SBP | 1.61 |
| Postoperative DBP | 1.57 |
| SIRI | 2.66 |
| NLR | 3.45 |
| PLR | 1.71 |
| SBP, systolic blood pressure; DBP, diastolic blood pressure; SIRI, systemic inflammation response index; NLR, neutrophil-to-lymphocyte ratio; PLR, platelet-to-lymphocyte ratio.  *For extent of resection, the generalized VIF scaled as GVIF^(1/(2·df)) is reported. | |

| **Table S3** **ROC analysis of inflammatory indices for postoperative hemorrhage** | | | | | | | | | | |
| --- | --- | --- | --- | --- | --- | --- | --- | --- | --- | --- |
| **Index** | **AUC (95%CI)** | **Cutoff** | **Sensitivity** | **Specificity** | **Youden** | **Accuracy** | **PPV** | **NPV** | ***P* value^Δ^** |  |
| SIRI | 0.686 (0.634-0.738) | 1.21 | 0.674 | 0.721 | 0.395 | 0.705 | 0.542 | 0.819 | / |  |
| NLR | 0.673 (0.620-0.726) | 3.05 | 0.597 | 0.707 | 0.305 | 0.671 | 0.500 | 0.782 | 0.369 |  |
| PLR | 0.669 (0.615-0.723) | 134.84 | 0.611 | 0.673 | 0.285 | 0.653 | 0.478 | 0.780 | 0.593 |  |
| ROC, receiver operating characteristic; AUC, area under receiver operating characteristic; CI, confidence interval; PPV, positive predictive value; NPV, negative predictive value; SIRI, systemic inflammation response index; NLR, neutrophil-to-lymphocyte ratio; PLR, platelet-to-lymphocyte ratio.  ^Δ^DeLong's test for AUC of SIRI vs NLR/PLR. | | | | | | | | | | |

| **Table S4 Incremental value of inflammatory indices for postoperative hemorrhage** | | | | | | | | | | | |  |
| --- | --- | --- | --- | --- | --- | --- | --- | --- | --- | --- | --- | --- |
| **Models** | **IDI** | |  | **cNRI** | |  | **Event NRI** | |  | **Non-Event NRI** | | |
|  | **Value (95% CI)** | ***P* value** |  | **Value (95% CI)** | ***P* value** |  | **Value (95% CI)** | ***P* value** |  | **Value (95% CI)** | ***P* value** | |
| Model 1 | / | / |  | / | / |  | / | / |  | / | / | |
| Model 1 + SIRI | 0.043 (0.023, 0.063) | < 0.001 |  | 0.657 (0.468, 0.847) | < 0.001 |  | 0.208 (0.049, 0.368) | 0.011 |  | 0.449 (0.347, 0.551) | < 0.001 | |
| Model 1 + PLR | 0.034 (0.016, 0.052) | < 0.001 |  | 0.518 (0.326, 0.710) | < 0.001 |  | 0.042 (-0.122, 0.205) | 0.617 |  | 0.476 (0.376, 0.577) | < 0.001 | |
| Model 1 + NLR | 0.021 (0.007, 0.036) | 0.004 |  | 0.539 (0.347, 0.731) | < 0.001 |  | 0.069 (-0.093, 0.232) | 0.404 |  | 0.469 (0.368, 0.570) | < 0.001 | |
| IDI, integrated discrimination improvement; cNRI, continuous net reclassification improvement; Event NRI, net reclassification improvement for patients with POH; Non-Event NRI, net reclassification improvement for patients without POH; SIRI, systemic inflammation response index; NLR, neutrophil-to-lymphocyte ratio; PLR, platelet-to-lymphocyte ratio.  Notes: All improvements calculated relative to Model 1 (basal ganglia location, tumor diameter, postoperative systolic blood pressure). | | | | | | | | | | | | |

| **Table S5 Specification of the final model** | | | | | |
| --- | --- | --- | --- | --- | --- |
| **Variable** | **Coding** | **Beta** | **SE** | **OR (95% CI)** | ***P* value** |
| Intercept | - | -7.7189 | 1.0384 | - | - |
| Basal ganglia location | 1 = basal ganglia, 0 = other | 0.6318 | 0.2627 | 1.881 (1.122-3.150) | 0.016 |
| Tumor diameter | per 1 cm | 0.2441 | 0.0723 | 1.277 (1.109-1.474) | <0.001 |
| Postoperative SBP | per 1 mmHg | 0.0315 | 0.0063 | 1.032 (1.020-1.045) | <0.001 |
| SIRI | per 1 unit | 0.4442 | 0.1414 | 1.559 (1.185-2.066) | 0.002 |
| PLR | per 100 units | 0.3988 | 0.1773 | 1.490 (1.056-2.122) | 0.025 |
| Final model fitted on the development cohort (n = 438). SBP, systolic blood pressure; SIRI, systemic inflammation response index; PLR, platelet-to-lymphocyte ratio; OR, odds ratio; CI, confidence interval; SE, standard error. | | | | | |

| **Table S6 Sensitivity analysis of the final model after sequentially excluding outliers of inflammatory indices** | | | | | |
| --- | --- | --- | --- | --- | --- |
| **Variables** | **Original model**  **(n = 438)** | **Model after excluding SIRI outliers (n = 426)** | **Model after excluding PLR outliers (n = 423)** | **Model after excluding NLR outliers (n = 416)** | **Model after excluding baseline hemorrhage (n = 400)** |
| SIRI | 1.56 (1.18-2.06, *P* = 0.002) | 1.84 (1.34-2.52, *P* < 0.001) | 1.69 (1.27-2.27, *P* < 0.001) | 2.44 (1.42-4.25, *P* = 0.001) | 1.48 (1.10–2.01, *P* = 0.011) |
| PLR (per 100 unit) | 1.49 (1.05-2.11, *P* = 0.025) | 1.71 (1.18-2.50, *P* = 0.005) | 2.21 (1.42-3.46, *P* < 0.001) | 2.91 (1.80-4.81, *P* < 0.001) | 1.69 (1.17–2.47, *P* = 0.005) |
| NLR | Not in final model | Not applicable | Not applicable | 0.82 (0.57-1.17, *P* = 0.282) | Not applicable |
| Data are presented as odds ratio (95% confidence interval, *P* value). SIRI, systemic inflammation response index; NLR, neutrophil-to-lymphocyte ratio; PLR, platelet-to-lymphocyte ratio.  The final model included tumor site (basal ganglia), tumor diameter, postoperative systolic blood pressure, SIRI, and PLR.  Outliers were defined using the boxplot method (values below Q1-1.5×IQR or above Q3+1.5×IQR).  For the “excluding NLR outliers” analysis, NLR was added to the model to assess whether it emerged as an independent risk factor.  The last column represents the analysis after excluding patients with hemorrhage identified on the baseline postoperative CT scan. | | | | | |

| **Table S7 Sensitivity analysis of the final model after adjustment for perioperative variables** | | | | | |
| --- | --- | --- | --- | --- | --- |
| **Variables** | **Final model (n = 438)** | |  | **Model with perioperative covariates (n = 438)** | |
|  | **OR (95% CI)** | ***P* Value** |  | **OR (95% CI)** | ***P* Value** |
| Basal ganglia location (vs other sites) | 1.88 (1.12-3.15 | 0.016 |  | 1.63 (0.95-2.81) | 0.078 |
| Tumor diameter, cm | 1.28 (1.11-1.47) | < 0.001 |  | 1.26 (1.08-1.47) | 0.004 |
| Postoperative SBP, mmHg | 1.03 (1.02-1.04) | < 0.001 |  | 1.03 (1.02-1.05) | < 0.001 |
| SIRI, per 1 unit | 1.56 (1.18-2.06) | 0.002 |  | 1.54 (1.16-2.05) | 0.003 |
| PLR, per 100 units | 1.49 (1.05-2.11) | 0.025 |  | 1.52 (1.07-2.17) | 0.021 |
| Extent of resection: subtotal vs gross total | – | – |  | 1.63 (0.91-2.92) | 0.104 |
| Extent of resection: partial vs gross total | – | – |  | 1.66 (0.76-3.64) | 0.205 |
| Surgical approach: endoscopic vs craniotomy | – | – |  | 0.70 (0.30-1.63) | 0.408 |
| Surgical approach: keyhole vs craniotomy | – | – |  | 0.89 (0.34-2.30) | 0.810 |
| Operative time, per hour | – | – |  | 0.99 (0.87-1.12) | 0.832 |
| Intraoperative blood loss, per 100 ml | – | – |  | 1.00 (0.92-1.10) | 0.928 |
| Intraoperative transfusion: > 400 vs ≤ 400 ml | – | – |  | 1.23 (0.45-3.33) | 0.686 |
| SBP, systolic blood pressure; SIRI, systemic inflammation response index; PLR, platelet-to-lymphocyte ratio. | | | | | |

| **Table S8 Analysis of interaction effects on postoperative hemorrhage** | | | |
| --- | --- | --- | --- |
| **Interaction Term** | **Adjusted OR (95% CI)** | ***P* Value** | **Likelihood Ratio Test *P* Value** |
| Interaction with clinical predictors |  |  |  |
| Basal ganglia × SIRI | 0.91 (0.49-1.68) | 0.765 | 0.767 |
| Tumor diameter × SIRI | 0.96 (0.82-1.12) | 0.577 | 0.578 |
| Postoperative SBP × SIRI | 1.00 (0.99-1.02) | 0.537 | 0.537 |
| Basal ganglia × PLR | 0.52 (0.25-1.07) | 0.077 | 0.076 |
| Tumor diameter × PLR | 1.05 (0.85-1.29) | 0.636 | 0.634 |
| Postoperative SBP × PLR | 1.01 (0.99-1.03) | 0.180 | 0.178 |
| Interaction with tumor type^*^ |  |  |  |
| Tumor type × SIRI | 0.58 (0.34-1.00) | 0.049 | 0.047 |
| Tumor type × PLR | 0.45 (0.22-0.89) | 0.022 | 0.019 |
| OR, odds ratio; CI, confidence interval; SIRI, systemic inflammation response index; PLR, platelet-to-lymphocyte ratio; SBP, systolic blood pressure.  PLR is scaled per 100 units.  *The exploratory interaction analysis with tumor type compared high-grade glioma versus all other tumor types; this categorization was adopted due to limited sample sizes within individual non-glioma subgroups. | | | |

| **Table S9 Univariable and multivariable analyses for clinically significant and surgically treated postoperative hemorrhage** | | | | | | |
| --- | --- | --- | --- | --- | --- | --- |
| **Variables** | **Univariate analysis** | |  | **Multivariate analysis** | |  |
|  | **OR (95% CI)** | ***P* value** |  | **OR (95% CI)** | ***P* value** | |
| **Clinically significant hemorrhage (n = 102)** | | | | | | |
| Basal ganglia location (vs other sites) | 1.59 (0.96-2.65) | 0.071 |  | 1.41 (0.82–2.43) | 0.219 | |
| Tumor diameter, cm | 1.43 (1.23-1.65) | <0.001 |  | 1.35 (1.16–1.57) | <0.001 | |
| Postoperative SBP, mmHg | 1.02 (1.10-1.03) | <0.001 |  | 1.02 (1.01–1.04) | <0.001 | |
| SIRI, per 1 unit | 1.61 (1.27-2.04) | <0.001 |  | 1.29 (0.97–1.71) | 0.082 | |
| PLR, per 100 units | 1.80 (1.32-2.44) | <0.001 |  | 1.45 (1.01–2.07) | 0.043 | |
| **POH requiring surgical reintervention (n = 37)** | | | | | | |
| Basal ganglia location (vs other sites) | 0.29 (0.09-0.97) | 0.045 |  | 0.27 (0.08-0.92) | 0.036 | |
| Tumor diameter, cm | 1.07 (0.87-1.31) | 0.532 |  | 1.03 (0.83-1.28) | 0.787 | |
| Postoperative SBP, mmHg | 1.03 (1.01-1.05) | 0.002 |  | 1.03 (1.01-1.05) | 0.003 | |
| SIRI, per 1 unit | 1.40 (1.01-1.94) | 0.046 |  | 1.24 (0.84-1.85) | 0.283 | |
| PLR, per 100 units | 1.66 (1.12-2.47) | 0.012 |  | 1.52 (0.94-2.44) | 0.088 | |
| OR, odds ratio; CI, confidence interval; SBP, systolic blood pressure; SIRI, systemic inflammation response index; PLR, platelet-to-lymphocyte ratio. | | | | | | |

# Supplementary Figures

**Supplementary Figure 1.** Restricted cubic spline analysis of the associations between inflammatory indices and postoperative hemorrhage. Adjusted odds ratios (solid line) with 95% confidence intervals (shaded area) are plotted for **(A)** SIRI and **(B)** PLR, modeled with restricted cubic splines (four knots) and adjusted for the final-model covariates. The associations were monotonically increasing but non-linear (non-linearity *P* < 0.001 for SIRI and *P* = 0.003 for PLR). The horizontal dashed line denotes an odds ratio of 1. SIRI, systemic inflammation response index; PLR, platelet-to-lymphocyte ratio.

**Supplementary Figure 2.** Distribution of inflammatory indices stratified by outlier status. Boxplots displaying the distribution of **(A)** SIRI, **(B)** NLR, and **(C)** PLR (stratified by outlier status: “No” = non-outlier; “Yes” = outlier). SIRI, systemic inflammation response index; NLR, neutrophil-to-lymphocyte ratio; PLR, platelet-to-lymphocyte ratio. Red dots represent outliers (defined as values < Q1 – 1.5 × IQR or > Q3 + 1.5 × IQR for each index)

**Supplementary Figure 3.** Calibration after outlier exclusion. Calibration of the final model after excluding outliers for **(A)** SIRI or **(B)** PLR. Overall performance remained stable, with C-index, calibration slope, intercept, and Brier score comparable to the primary analysis. In the development cohort, the Hosmer–Lemeshow test indicated adequate calibration under both conditions (*P* = 0.422 and *P* = 0.648, vs *P* = 0.029 in the primary analysis); the validation cohort maintained adequate calibration (*P* > 0.05). Metrics are based on the corresponding outlier-excluded subsample. SIRI, systemic inflammation response index; PLR, platelet-to-lymphocyte ratio.
